# Supplementary material for: Planar carbon nanotube–graphene hybrid films for high-performance broadband photodetectors
Source: Nat Commun. 2015 Oct 8;6:8589. doi: 10.1038/ncomms9589 (PMC4633958; doi:10.1038/ncomms9589)
Supplement: Supplementary Information — Supplementary Figures 1-8, Supplementary Notes 1-3 and Supplementary References [file ncomms9589-s1.pdf]

SUPPLEMENTARY FIGURES

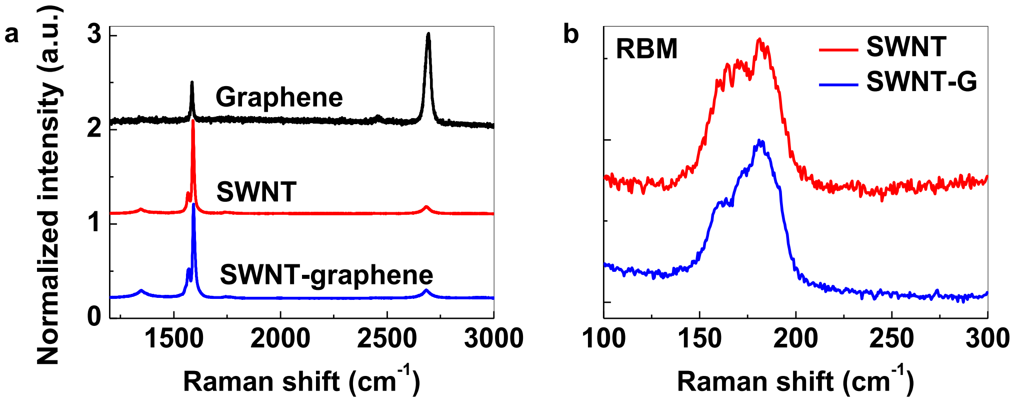

Supplementary Figure 1. Raman spectrum. a, for graphene, SWNTs, and SWNT-graphene hybrid, respectively. The Raman spectra are offset for clarity. **b**, the zoomed-in Raman spectra of the RBM.

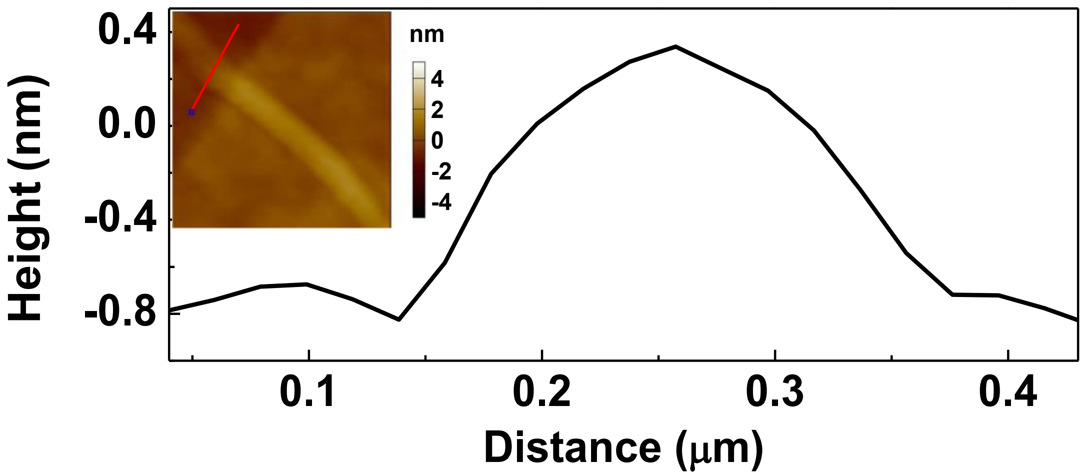

Supplementary Figure 2. Height profile of the SWNT in Figure 1c. It is observed that this single-walled nanotube is with a diameter of about 1.2 nm. Inset shows the red line along which the height profile of the SWNT was measured.

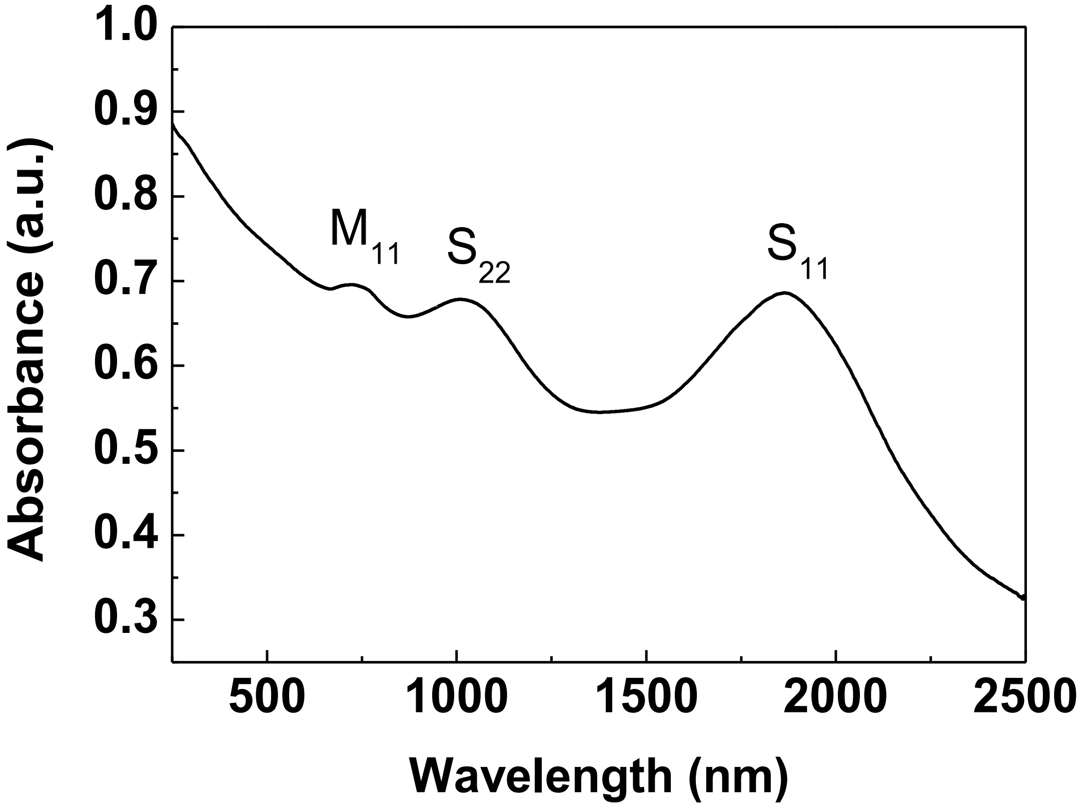

Supplementary Figure 3. Optical absorbance spectrum of SWNTs with high loadings. The excitonic absorption peaks corresponding to the optical transitions associated with the van Hove singularities in the density of states of SWNTs can be observed. The peaks at ~1800 nm and ~1000 nm correspond to the S_{11} and S_{22} exciton transitions in semiconducting SWNTs respectively. The peak at ~ 700 nm corresponds to the M_{11} exciton transitions in metallic SWNTs.

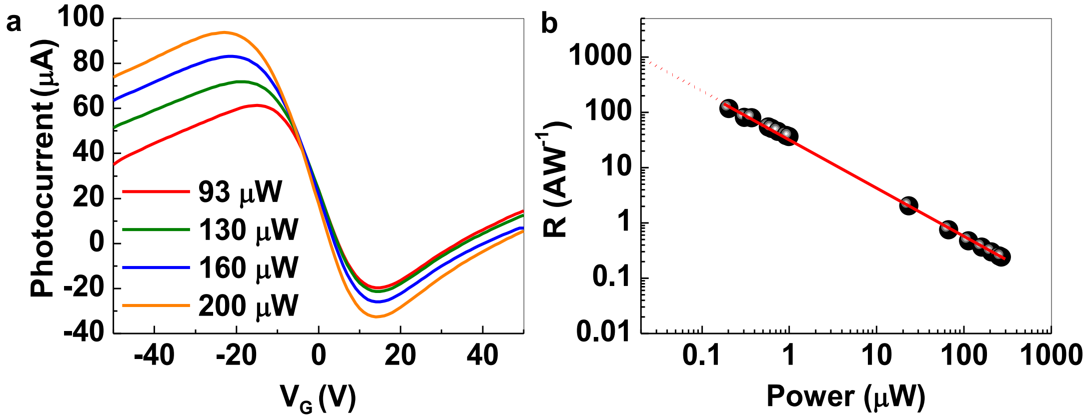

Supplementary Figure 4. a, Gate voltage-dependent photocurrent at 650 nm illumination ($I_{ph}=I_{dark}-I_{light}$). **b**, Responsivity versus optical illumination power. The red line is the linear fit to the data, showing an inferred responsivity of ~1000 AW^{-1} at ~0.01 μW.

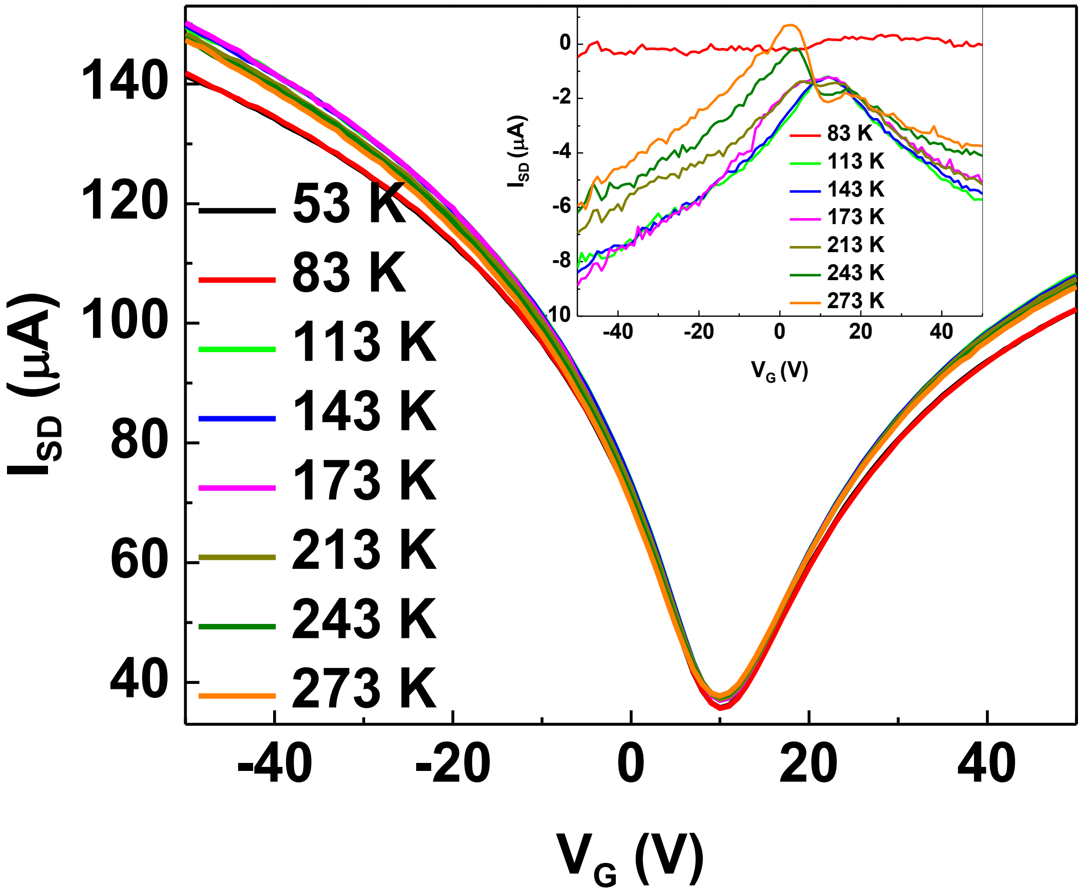

Supplementary Figure 5. I_{SD} dependence of sample temperature when cooling down from room temperature (V_{SD}=0.5 V). Inset: temperature-dependent change in source-drain current from 53 K value ($\Delta I_{SD}=I_T-I_{53\ K}$) as a function of back-gate voltage.

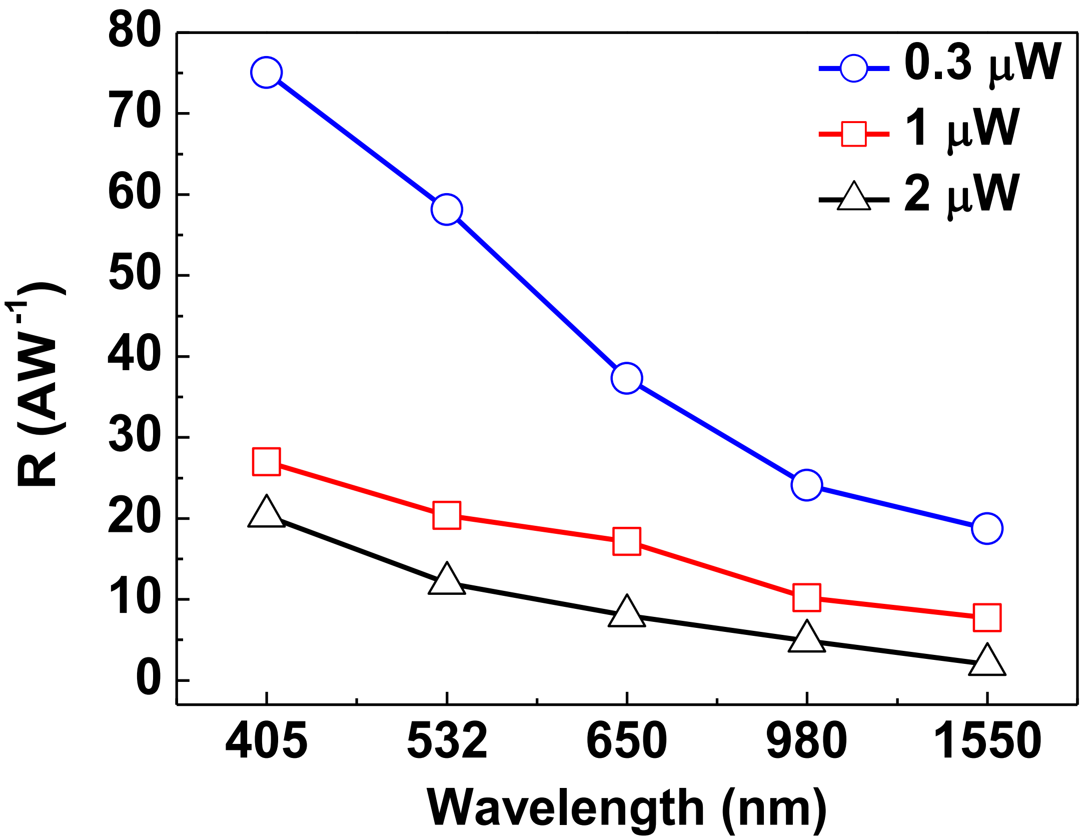

Supplementary Figure 6. Ultra-broadband (405 nm – 1550 nm) photoresponsivities for different illumination power of 0.3, 1 and 2 μW. V_{SD}=0.5 V.

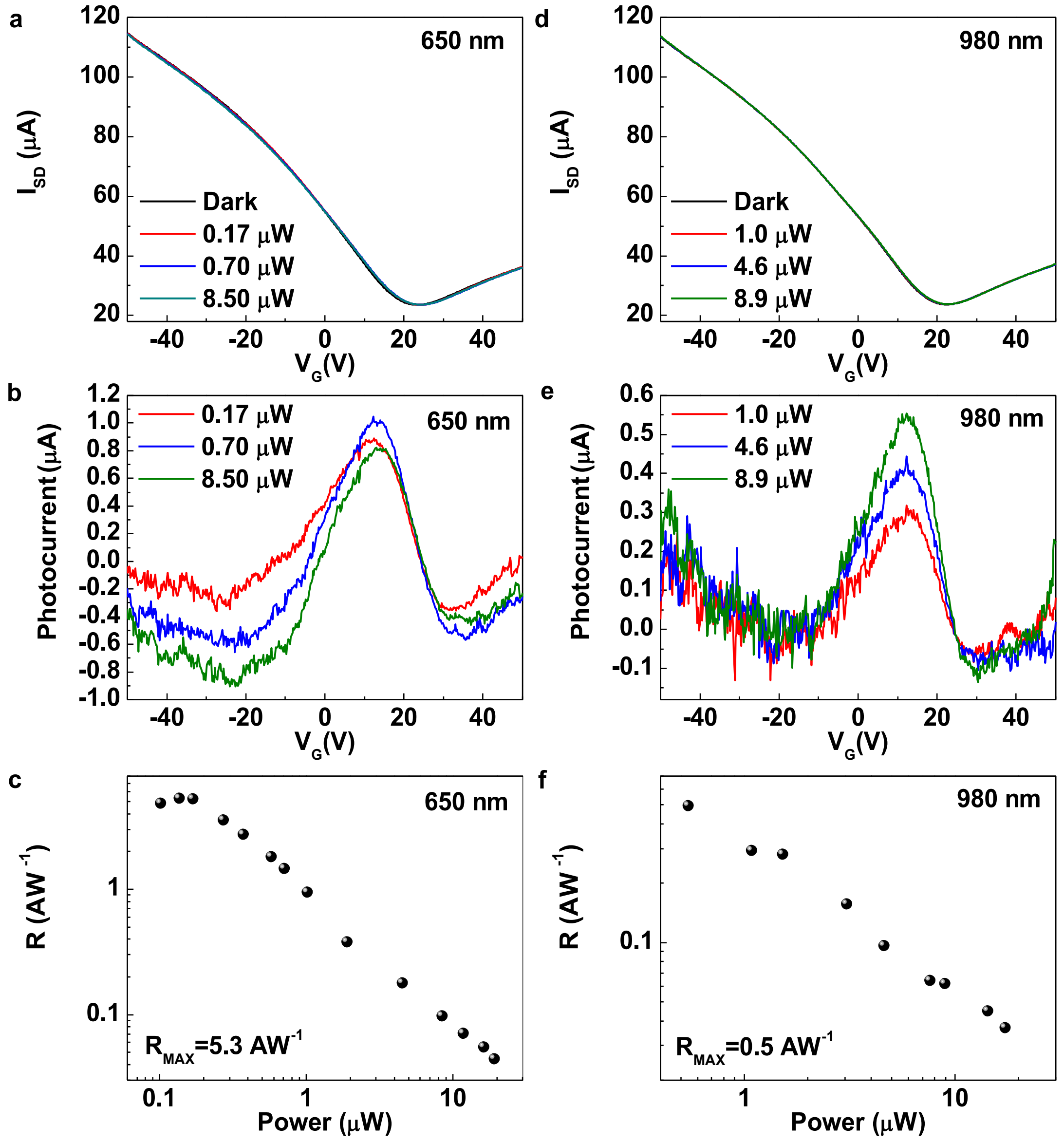

Supplementary Figure 7. Photoresponse performance of metallic SWNT-graphene phototransistor under 650 nm and 980 nm illumination. (The purity of the metallic SWNTs is >99%.) **a**, Source-drain current (I_{SD}) as a function of back-gate voltage (V_G) with increasing 650 nm illumination intensities. V_{SD}=0.5 V. It is found that the transfer curve shifts toward positive V_G with increasing laser power, and the Dirac point voltage shifts to the right. These observations indicate that electrons transfer from graphene to metallic SWNTs, which is opposite to the effect observed for semiconducting SWNTs based hybrid devices. **b,** Gate voltage-dependent photocurrent at 650 nm illumination. The net photocurrent is obtained by subtracting the dark current from the light current. **c**, Responsivity as a function of the 650 nm illumination power. The responsivity saturates at about 5.3 AW^{-1} and decreases with increasing power. **d**, Source-drain current (I_{SD}) as a function of back-gate voltage (V_G) for the metallic SWNT-graphene device with increasing 980 nm

illumination intensities. V_{SD}=0.5 V. **e**, Gate voltage-dependent photocurrent at 980 nm illumination. **f**, Responsivity as a function of the 980 nm illumination power.

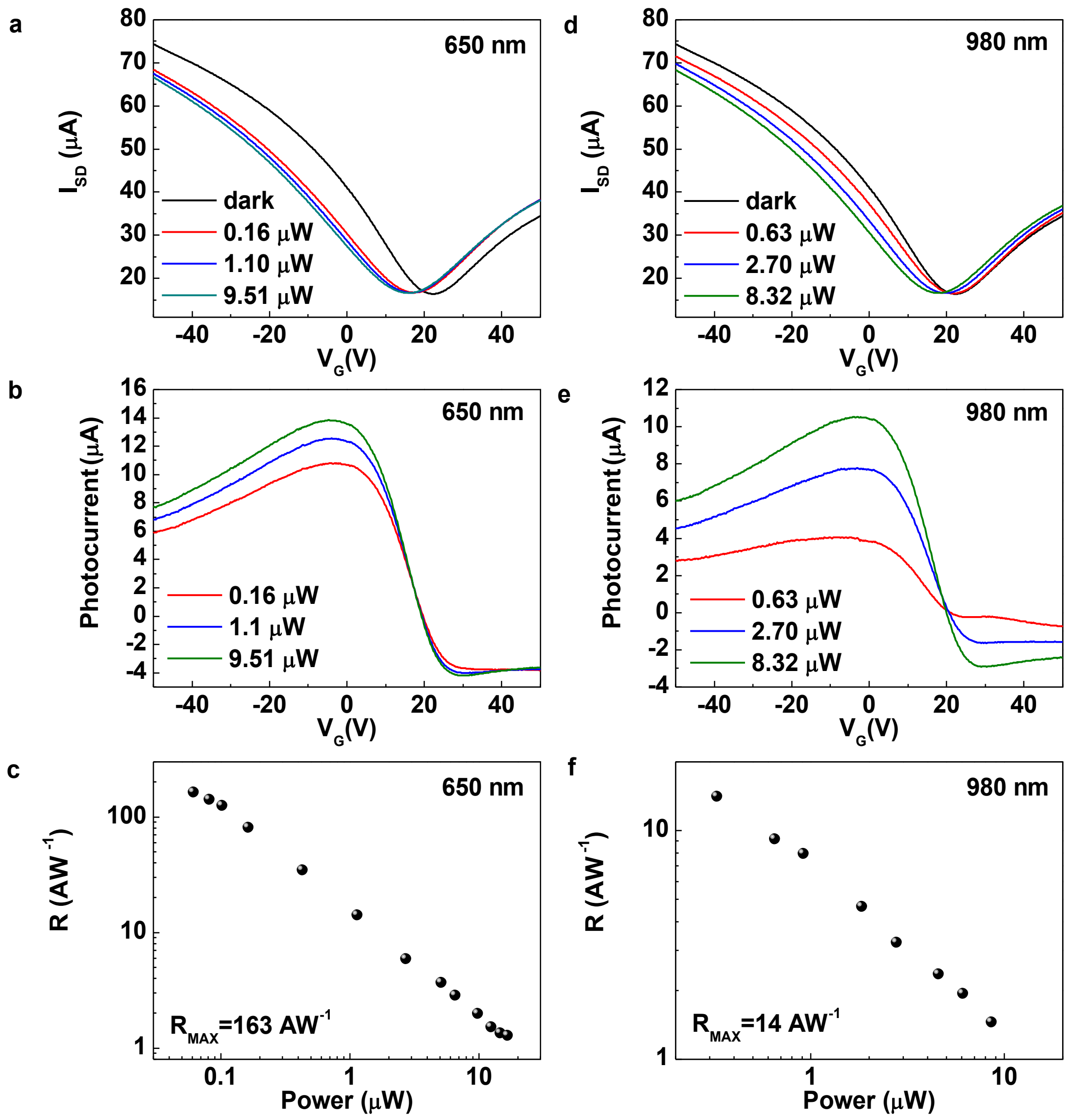

Supplementary Figure 8. Photoresponse performance of semiconducting SWNT-graphene phototransistor under 650 nm and 980 nm illumination. (The purity of single chirality (6, 5) semiconducting SWNTs is >93%.) **a**, Source-drain current (I_{SD}) as a function of back-gate voltage (V_G) with increasing 650 nm illumination intensities. V_{SD}=0.5 V. It is observed that with increasing illumination power, the Dirac point voltage shifts to the left. **b,** Gate voltage-dependent photocurrent at 650 nm illumination. **c**, Responsivity as a function of the 650 nm illumination power. The responsivity of the high-purity semiconducting SWNT-graphene phototransistors are in the same order with our previous results, indicating that the photoresponse is mainly from the semiconducting SWNTs, and the contribution from the metallic SWNTs is negligible. **d**, Source-drain current (I_{SD}) as a function of back-gate voltage (V_G) with increasing 980 nm illumination intensities. V_{SD}=0.5 V. **e**, Gate voltage-dependent photocurrent at 980 nm illumination. **f**, Responsivity as a function of the 980 nm illumination power.

SUPPLEMENTARY NOTES

Supplementary Note 1. Analysis of the Raman signatures

Raman spectra were used to confirm the single-layer feature of the graphene using 514 nm excitation (1 mW). Supplementary Figure 1 shows the Raman spectrum of graphene, SWNTs layer and SWNT-graphene hybrid, respectively. The characteristic Raman modes[1] of SWNTs are the RBM (radial breathing mode) (100-300 cm^{-1}), the G band (1500-1700 cm^{-1}), the D band (1300-1400 cm^{-1}) and the 2D band (2600-2700 cm^{-1}). Due to the curvature of the SWNTs, the G peak splits into two components G^- (at about 1570cm^{-1}) and G^+ (at about 1592 cm^{-1}). The black curve in Supplementary Figure 1a is a typical Raman spectrum of graphene[2]. The absence of the D peak indicates the defect-free feature of the graphene sample. The 2D peak can be well fitted by a symmetric and sharp Lorentzian peak with FWHM (Full Width at Half Maximum) ~ 28 cm^{-1}, signature of single layer graphene. The G peak position and FWHM are ~ 1587 cm^{-1} and ~ 9 cm^{-1}, respectively. The peak intensity ratio I_{2D}/I_G ~ 2 and the area ratio A_{2D}/A_G ~ 8.2 suggest that the graphene sheet is monolayer with reasonably good quality. From the Radial Breathing Mode (RBM) of the SWNTs (Supplementary Figure 1b), the mean diameter of the SWNTs is estimated[3] to be 1.4 nm, consistent with the AFM results.

Supplementary Note 2. Temperature dependent transfer characteristics

Supplementary Figure 5 shows the change in transport current compared to the 53 K value when cooling down in the cryostat for one typical SWNT-graphene hybrid phototransistor. We can see that the source-drain current (I_{SD}) increases (or the resistance decreases) as the temperature increases from 53 K to 173 K. I_{SD} decreases slightly as the temperature increases from 173 K to 273 K, but is still much higher than that of 53 K. The temperature dependence of the ΔI_{SD} (I_T-I_{53K}) is shown in the inset of Supplementary Figure 5. Taken together, the characteristics clearly rule out thermal effects as origin of the photoresponse.

Supplementary Note 3. The influence of SWNTs' electronic type, chirality and diameter on the photoresponse

We fabricated control devices based on different kinds of SWNTs samples, i.e. metallic and semiconducting SWNTs. As shown in Supplementary Figure 7, ultra-high purity (>99% purity) metallic SWNTs were studied with 650 nm and 980 nm wavelength excitation. Only small photoresponsivities (about 5% of those in semiconductor SWNTs) were observed. Notably, in contrast to the semiconducting SWNTs case, the photogating polarity is also inversed, and the responsivity saturates eventually at very small input power. The results could be understood by considering hot-carrier injection from graphene to SWNTs. Due to the metallic nature of SWNTs, the build-in potential at the graphene/SWNTs junction is effectively screened, resulting in very weak electron-hole pair separation and charge transfer from SWNTs to graphene. In this scenario, the

photo-carrier generation is determined by light absorption in graphene, i.e. hot-electrons in graphene are injected into SWNTs. The rapidly saturated photoresponse and the p-type photogating effect agree well with this interpretation.

We also repeated our measurements by using (6, 5) chirality enriched semiconducting SWNTs (>93% purity). As shown in Supplementary Figure 8, the responsivities does not change much compare with our results based on highly purified SWNTs ensembles. This suggests that the distribution of tube diameters or chiralities for a given electronic type, i.e. semiconducting, only slightly affects the photoresponse.

In conclusion, the electronic type of SWNTs determines the photodetection mechanism. In highly purified SWNTs networks, such as the sample used in our manuscript, the contribution from metallic SWNTs is negligible.

SUPPLEMENTARY REFERENCE

1. Paulus G. L. C., *et al.* Charge Transfer at Junctions of a Single Layer of Graphene and a Metallic Single Walled Carbon Nanotube. *Small* **9**, 1954-1963 (2013).
2. Ferreira E. H. M., *et al.* Evolution of the Raman spectra from single-, few-, and many-layer graphene with increasing disorder. *Physical Review B* **82**, 125429 (2010).
3. Strano M. S., Doorn S. K., Haroz E. H., Kittrell C., Hauge R. H., Smalley R. E. Assignment of (n, m) Raman and optical features of metallic single-walled carbon nanotubes. *Nano Lett.* **3**, 1091-1096 (2003).
